# Hydrodynamics of Droplet Sorting in Asymmetric Acute Junctions

**DOI:** 10.3390/mi13101640

**Published:** 2022-09-29

**Authors:** He Yang, Tuomas P. J. Knowles

**Affiliations:** 1 School of Mechanical Engineering, Hangzhou Dianzi University, No. 2 Street, Qiantang District, Hangzhou 310018, China; 2 Yusuf Hamied Department of Chemistry, University of Cambridge, Lensfield Road, Cambridge CB2 1EW, UK; 3 Cavendish Laboratory, University of Cambridge, JJ Thomson Avenue, Cambridge CB3 0HE, UK

**Keywords:** microfluidics, droplet sorting, asymmetric junction, passive sorting, branching channel

## Abstract

Droplet sorting is one of the fundamental manipulations of droplet-based microfluidics. Although many sorting methods have already been proposed, there is still a demand to develop new sorting methods for various applications of droplet-based microfluidics. This work presents numerical investigations on droplet sorting with asymmetric acute junctions. It is found that the asymmetric acute junctions could achieve volume-based sorting and velocity-based sorting. The pressure distributions in the asymmetric junctions are discussed to reveal the physical mechanism behind the droplet sorting. The dependence of the droplet sorting on the droplet volume, velocity, and junction angle is explored. The possibility of the employment of the proposed sorting method in most real experiments is also discussed. This work provides a new, simple, and cost-effective passive strategy to separate droplets in microfluidic channels. Moreover, the proposed acute junctions could be used in combination with other sorting methods, which may boost more opportunities to sort droplets.

## 1. Introduction

Droplet-based microfluidics has received increasing interest over the recent two decades, due to the large amount of applications in chemical reactions [1,2], materials synthesis [3,4], protein engineering [5,6,7,8], drug discovery [9,10,11], and single-cell analysis [12]. As one of fundamental operations in droplet-based microfluidics, droplet sorting based on various contents is used to achieve the enrichment, isolation, and quantitative analysis of the samples. The ability to sort droplets is of crucial importance to chemical and biological applications, such as microreactors [13], drug screening [14,15], and cell encapsulation [16]. Therefore, there is an enormous demand to rationally design and develop sorting methodology for the vast applications of the droplet-based microfluidics.

Many methods have been proposed to achieve droplet sorting in the droplet-based microfluidics, both actively and passively. The active sorting methods mainly include electrical sorting, acoustic method, magnetic control, pneumatic actuators, and thermal methods [17,18]. The electric sorting uses electric forces to push or pull droplets with charges. The early attempt of using electric control was made by Link et al. [19]; the droplets are sorted into the collective channel under the effect of electrostatic forces caused by the direct current. In this method, pre-charged droplets are required before they are sorted, which can be realized by exposing the droplets to energized electrodes [20,21] or deploying the induction effect of electric fields [22,23]. Another electric method for droplet sorting is based on the dielectrophoresis effect of alternating current, which produces dielectric forces on the droplets [24,25,26,27]. Electric control usually requires a high voltage to activate the electrodes [13,20,27]. Acoustic sorting is based on the acoustic streaming or acoustic radiation forces impacting on the droplets. Three kinds of acoustic modes are developed to sorting the droplets, including travelling surface acoustic [28,29,30], standing surface acoustic wave [31,32,33,34], and bulk acoustic wave [35]. Although the acoustic methods have good sensitivity, accuracy, throughput, contactless operation, and biocompatibility, they require expensive equipment to operate, including function generators, voltage amplifiers, signal processors, and acoustic transducers [36]. Magnetic sorting is based on the magnetic forces on the droplets exerted by electromagnet or permanent magnet. Usually, ferrofluid, magnet particles, or beads are used in the continuous phase or dispersed phase to fulfil magnet sorting [37,38,39,40]. The drawback of the magnetic sorting is that the required magnetic materials limit their biological or chemical applications. It is impossible to remove the magnetic labels for downstream analysis and the labels may change the properties of the cells [18]. Pneumatic sorting uses the deformation of the microchannels or microvalves to adjust the hydrodynamic pressure or resistance [41,42]. The pneumatic methods have a good flexibility of droplet size, channel geometry, chemical formulation, and biocompatibility but relatively low durability and throughput [17]. Thermal sorting is based on the thermocapillary effect produced by additional resistive heating [43] or focused laser heating [44]. The thermocapillary forces and the change in the fluid viscosity and interfacial tension deflect the droplets to the collective channels, achieving droplet sorting [17,45]. The predominant advantage of active sorting methods is that they can realize on-demand control of the droplet when incorporating the detection methods. However, these methods require additional energy input and the complex structure of the active sorting would increase the fabrication difficulty and the cost, especially for those high-throughput chips containing hundreds and thousands of microfluidic channels.

Passive sorting methods have the advantages of easy fabrication, low cost, biocompatibility, and no requirement of external control components. In principle, the passive droplet sorting usually adopts the hydrodynamic features to manipulate the droplets [46]. Many passive methods have been proposed over the last two decades, including grooves [47,48], inertial microfluidics [49], deterministic lateral displacement [50], and microscale filters [51]. Droplet sorting with groove rails is based on the Laplace pressure. When the droplets encounter the parallel rails, they are trapped and follow the rail path. The small droplet is maintained in the old grooves, while the large droplets are transferred to new rails under Laplace pressure [52]. The inertial microfluidics uses the inertial lift forces to separate the droplets. Specifically, droplets of different sizes or deformability have different lateral equilibrium positions under the lift forces [53,54]. The averaged lateral position of the large droplet is closer to the channel centreline than that of the small one [49]. The deterministic lateral displacement relies on the hydrodynamic forces. The droplets of various sizes or deformability displace differently in the lateral direction [55,56,57,58]. The microscale filters separate droplets based on the size exclusion [51]. The size of the passive filter needs to be precisely controlled to filter the desired droplets. Although these passive methods have already been investigated, new passive methods are still required to be developed to achieve the simple, cost-effective, high-throughput, and precise sorting of the droplets for extensive biochemical applications.

Asymmetric microchannels have attracted the interest of many researchers, due to their unique manipulation of the droplet breakup. A simple asymmetric junction can be achieved by varying the length or width of the T junction [59]. This asymmetric T junction is used for producing unequal-sized daughter droplets. The length ratio of the daughter droplets depends on the capillary number, initial droplet length, length ratio, and width ratio of the branching channels [60,61,62]. Another kind of asymmetric junction is that the bifurcating channels are intersected with a non-rectangular angle. Ménétrier-Deremble and Tabeling [63] examined droplet breakup behavior in the asymmetric junctions with an angle of 45° and 135°. Both direct and retarded breakup process were observed. The breakup behavior is strongly affected by the channel geometry. Wang et al. [64] investigated an asymmetric splitting junction, in which two channels are intersected with an angle of 30°. The splitting ratio of daughter droplets is largely affected by the initial droplet length and the velocity. Although a number of studies are reported to explore the droplet flow in asymmetric microchannels, they mainly focus on the asymmetric splitting of the droplets, rather than droplet sorting behavior.

In this work, we investigate whether the droplet sorting can be achieved using asymmetric acute junctions. Both volume-based and velocity-based sorting are numerically investigated using the volume of fluid (VOF) model. The effect of junction angle on the sorting behavior is explored in detail. The angle of the acute junction varies from 20° to 80°. The numerical details are described in Section 2. Section 3 presents the volume-based and velocity-based sorting and then a phase regime of the droplet sorting is presented to illustrate the dependence of the sorting behavior on the junction angle, droplet volume, and droplet velocity. Section 4 discusses whether the proposed sorting method can be used in most real applications of droplet-based microfluidics. Finally, the main conclusions are drawn in Section 4.

## 2. Numerical Details

### 2.1. Geometry of the Asymmetric Acute Junction

**Figure 1:**
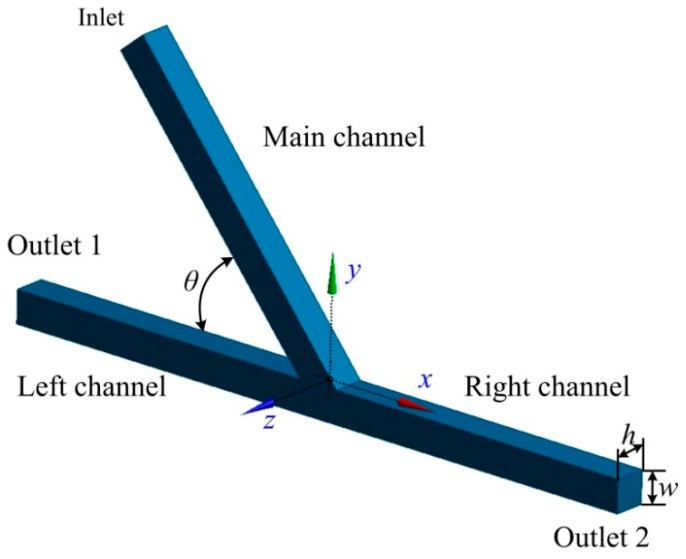
Geometry of the asymmetric acute junction.

The geometry of the asymmetric microfluidic junction includes the main, left, and right channels, as shown in Figure 1. The main and left channels are intersected with an acute angle *θ*, while the left and right channel are aligned. All three channels have a width of *w* = 100 μm and a depth of *h* = 100 μm. The main channel has a length of *L*_m_ = 11 *w*, while the two branching channels have the same length of *L*_L_ = *L*_R_ = 10.5 *w*. The numerical results shows that these dimensions are long enough to obtain an equilibrium of the droplet shape before arriving at the acute junction. Water-in-oil droplets were investigated in this work, that is, the droplet fluid and the medium fluid were set to water and oil, respectively. Following previous work conducted by Hoang et al. [65], the viscosity and density of the water droplet were *μ*_d_ = 1 × 10^−3^ Pa·s and of *ρ*_d_ = 1000 kg/m^3^, respectively. The medium oil fluid had a viscosity of *μ*_m_ = 8 × 10^−3^ Pa·s and a density of *ρ*_m_ = 770 kg/m^3^. The interfacial tension between the medium fluid and droplet fluid was *σ* = 5 × 10^−3^ N/m. The velocity *U* of the main channel varied from 4 × 10^−3^ to 4 × 10^−2^ m/s, and the corresponding capillary number was *Ca* = *μ*_m_*U/σ* = 6.4 × 10^−3^–6.4 × 10^−2^. The capillary number represents the relative effect of viscous force versus surface tension. The non-dimensional volume of the droplet was within the range of *V*_d_/*w*^3^ = 0.5–2.5.

### 2.2. Governing Equations

In this work, we used the volume of fluid (VOF) model [66] to simulate the interface between the droplet and the medium fluid, which has been successfully applied to modelling various droplet flows (e.g., Refs. [67,68,69]). The VOF model is a numerical method for modelling the liquid–liquid interface. It is based on the use of a fractional function C, which is defined as an integral of the characteristic functions of the fluid in a computational grid cell. C is a continuous function, with 0 ≤ C ≤ 1. If the cell is empty of the tracked fluid, C = 0. If the cell is full of tracked fluid, C = 1. Additionally, if the cell contains an interface between the tracked and non-tracked fluids, 0 < C < 1. The governing equations for the droplet flow in microfluidic channel consist of a continuity equation and momentum equation, which can be expressed as follows,

(1) $$\frac{\partial \rho }{\partial t}+\nabla \cdot \left(\rho u\right)=0$$

(2) $$\frac{\partial (\rho u)}{\partial t}+\nabla \cdot \left(\rho uu\right)=-\nabla p+\nabla \cdot [\mu (\nabla u+{\left(\nabla u\right)}^{T})]+\rho g+F_{s}$$

where *p* is the pressure, and *ρ* and *μ* represent the density and viscosity of the two-phase fluids, respectively. **F_s_** denotes the surface tension force obtained by the continuum surface force (CSF) model [70]. The density, viscosity, and surface tension force of the two-phase fluids can be calculated by

(3) $$\rho =\alpha_{d}\rho_{d}+\alpha_{m}\rho_{m}$$

(4) $$\mu =\alpha_{d}\mu_{d}+\alpha_{m}\mu_{m}$$

(5) $$F_{s}=\gamma \frac{\rho \kappa_{d}\nabla \alpha_{d}}{\frac{1}{2}(\rho_{c}+\rho_{d})}$$

where *ρ_d_* and *ρ_m_* represent the fluid density of the droplet and medium phase, respectively. *μ_d_* and *μ_c_* denote the fluid viscosity of the droplet and medium phase, respectively. *α_d_* and *α_m_* are the volume fraction of the droplet and medium phase, respectively. *α_d_* + *α_m_* =1. *κ_d_* is the curvature of the phase interface.

The numerical simulations were conducted using commercial software Fluent 17.0. The transient laminar flow and VOF model were used for the calculations of the governing equations. Velocity-inlet and pressure-outlet boundary conditions were applied for the inlet of the main channel and the outlets of the left and right channels, respectively. A no-slip stationary boundary condition was employed at the walls, which were assumed to be super hydrophobic with a contact angle of 180°, as was conducted in Refs [65,67]. The SIMPLEC method was used to calculate the pressure–velocity coupling, due to the less convergence time [60,71]. Following previous studies (e.g., Refs. [67,68,69]), the Geo-Reconstruct scheme was chosen to solve the volume fraction of the two immiscible fluids and the PRESTO! algorithm was applied to calculate the pressure term. The second order upwind scheme was used to calculate the momentum term. The grid independence study was performed, which shows that the grid size of *L*_grid_ = 1/30 *w*, where *w* is the channel width, was enough to obtain the satisfactory results. Numerical simulations were conducted on a workstation (Intel^®^Xeon^®^ Platinum 8358P with dual processors, 32 cores, and 256 GB RAM). The computation time for each run was approximately 2 to 4 days, which varied at different inlet velocity.

### 2.3. Model Validation

Previous studies (e.g., Ref. [65]) have successfully proven that VOF model can be used to simulate the droplet formation and breakup in microfluidic channel. In this work, the numerical model was further validated by examining the Laplace’s law and Taylor deformation of the droplet in microfluidic channels, as was conducted in Ref. [72].

**Figure 2:**
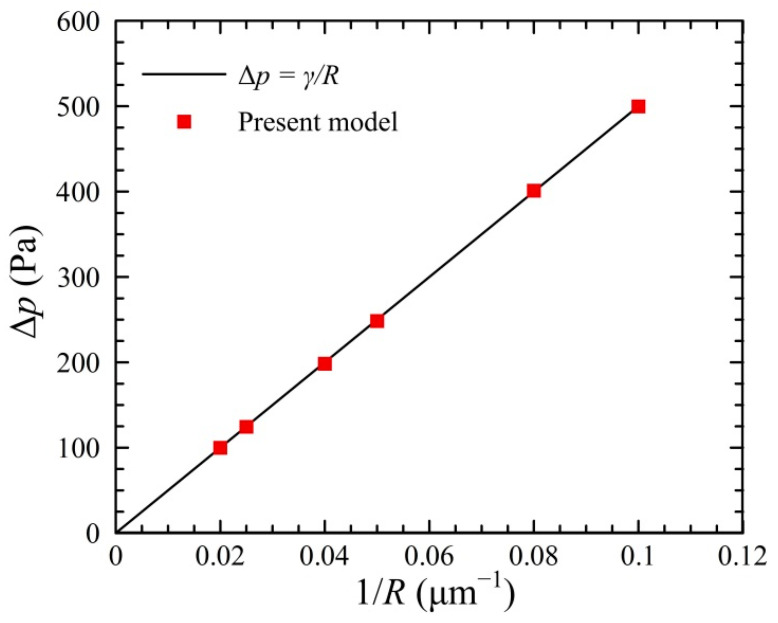
Numerical validation of Laplace’s law.

We first validated our model by examining the Laplace’s law. A circular water droplet was located at the center of a square oil domain with the size of 256 μm × 128 μm. The mesh had a grid size of Δ = 1 μm. No slip stationary wall conditions were applied to all boundaries. In this simulation, the water had a density of *ρ*_w_ = 1000 kg/m^3^ and a viscosity of *μ*_w_ = 1 × 10^−3^ Pa·s, while the oil had a density of *ρ*_d_ = 1000 kg/m^3^ and a viscosity of *μ*_o_ = 8 × 10^−3^ Pa·s. The interfacial tension of two fluid was *γ* = 5 × 10^−3^ N/m. The pressure difference Δ*p* across the droplet interface in the equilibrium state was provided by the Laplace’s law Δ*p = γ/R*, where *R* denotes the droplet radius. It can be observed from Figure 2 that the pressure difference Δ*p* exhibits a linear relationship with the 1/*R*, and the numerical results of present model agree well with those calculated by the Laplace’s law. This indicates that the present model is acceptable for predicting the droplet size in the microchannel.

To further validate the numerical model, a three-dimensional (3D) deformation of the droplet in a simple shear flow was examined. In this case, the computational domain had a length of *L_s_* = 128 μm and a width and a height of *W_s_* = *H_s_* = 62 μm. The initial diameter of the droplet was set to be *D_i_* = 20 μm. The density and the viscosity of droplet and medium fluids were *ρ_d_ = ρ_m_* = 1000 kg/m^3^ and *μ_d_ = μ_m_* = 1 × 10^−3^ Pa·s. The capillary number *Ca* varied from 0 to 0.03. The three-dimensional deformation *D* of a droplet in a simple shear flow was calculated using the Taylor deformation number [73],

(6) $$D=\frac{L-B}{L+B}=\frac{19\mu_{d}+16\mu_{m}}{16\mu_{d}+16\mu_{m}}=\frac{35}{32}Ca$$

where *L* and *B* denote the major and minor axes of the deformed droplet, respectively.

**Figure 3:**
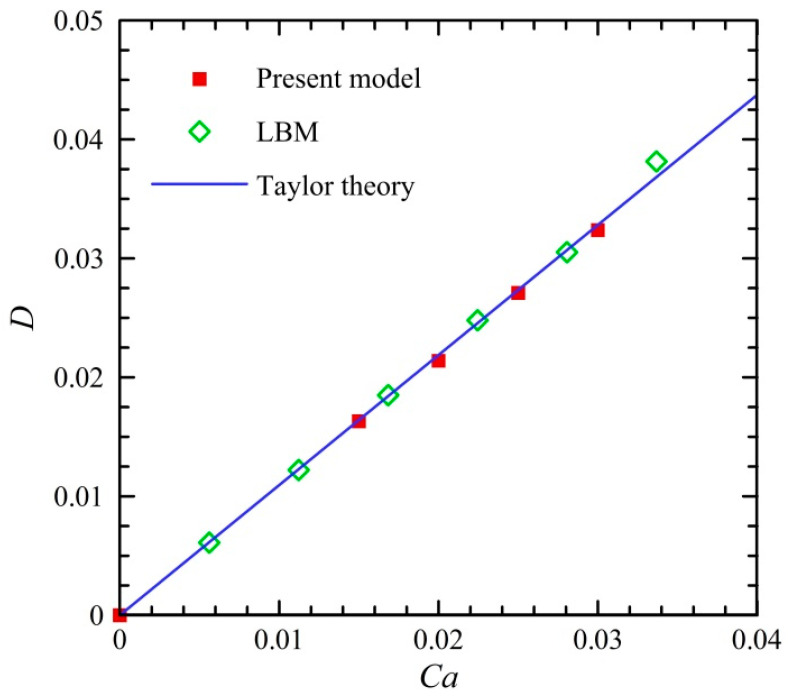
Droplet deformation in a simple shear flow.

Figure 3 presents the dependence of droplet deformation in the shear flow on the capillary number. The results of droplet deformation simulated by the Lattice Boltzmann method (LBM) [73] are shown for the comparison. It can be observed that the present results agree well with those obtained by the theoretical prediction and the LBM. This indicates that present model can be used for the modelling of the deformed droplet within a satisfactory accuracy.

## 3. Results

### 3.1. Volume-Based Droplet Sorting

The asymmetric microfluidic junction with an acute angle between the main channel and the branching channel can be used for volume-based droplet sorting. Large droplets are sorted into the left channel, while small droplets enter into the right channel. Both the subsequent stages of the droplet sorting and corresponding pressure distributions are examined in this section.

**Figure 4:**
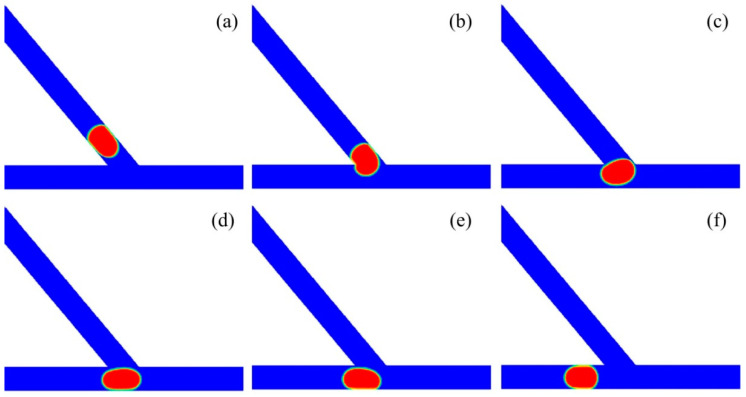
Passive sorting of large droplet into the left channel (*θ* = 50°, U = 20 mm/s, *V*_d_*/w*^3^ = 0.85). (**a**) T0, (**b**) T1 = T0 + 0.8, (**c**) T2 = T0 + 1.4, (**d**) T3 = T0 + 1.8, (**e**) T4 = T0 + 2.8, (**f**) T5 = T0 + 4.8.

Figure 4 shows the subsequent stages of large droplet sorting into the left channel of the asymmetric junction. The time propagates from T0 to T5, with the time intervals rescaled by *w/U*. The angle of the asymmetric junction was fixed at *θ* = 50°. The non-dimensional volume of the droplet was *V*_d_*/w*^3^ = 0.85 and the inlet velocity as fixed at *U* = 20 mm/s, with the corresponding capillary number of *Ca* = 0.032. Initially, the droplet starts to move into the junction at the time of T0. At T1, the droplet enters into the junction earlier at the left side than at the right side, due to the asymmetric structure of the junction. As a result, the head of the droplet turns left slightly under the effect of the Laplace pressure. Then, the droplet moves towards the bottom wall of the branching channel, and the rear of the droplet becomes flat under the push effect of the upstream fluid. At T4, the droplet starts to move into the left channel, and there is a tunnel between the right part of the droplet and the right corner of the junction. As such, the medium fluid mainly travels towards the right channel through this tunnel. Finally, the droplet completely enters into the left channel at T5.

**Figure 5:**
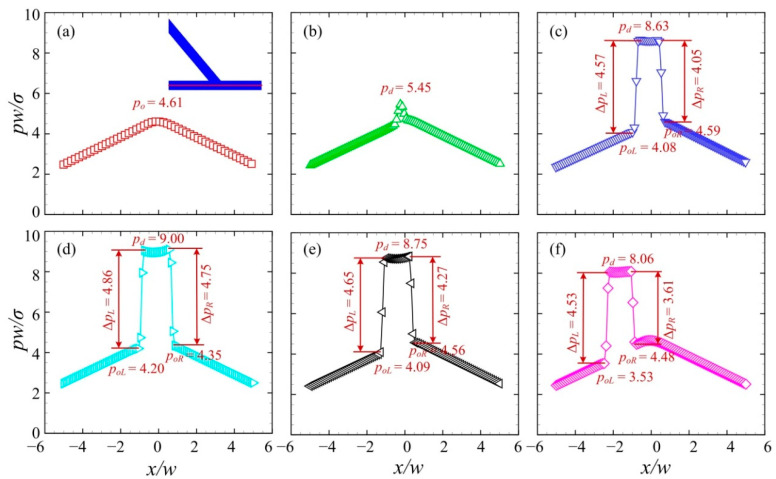
Pressure distributions along the centreline of the branching channels at a droplet volume of *V*_d_*/w*^3^ = 0.85 (*θ* = 50°, *U* = 20 mm/s). (**a**) T0, (**b**) T1, (**c**) T2, (**d**) T3, (**e**) T4, (**f**) T5.

Figure 5 presents the pressure distributions along the centreline of the branching channels. The pressure *p* is rescaled by *σ/w*. Six sequential times were chosen, corresponding to those in Figure 4. *p_d_* and *p_o_* denote the pressure of the water droplet fluid and the oil medium fluid, respectively. *p_oL_* and *p_oR_* represent the pressure of the medium oil fluid on the left and right of the droplet, respectively. At T0, the pressure exhibits a hill-like distribution with the largest value at the center. At T1, the pressure at the central region starts to rise, indicating that the droplet begins to move into the asymmetric junction. Further at T2, the pressure of the droplet increases substantially to *p_d_* = 8.63. Δ*p_L_* and Δ*p_R_* represent the pressure difference across the left and right interfaces, respectively. Correspondingly, the curvature radius *R_L_* and *R_R_* of the left and right interfaces are determined by Laplace’s law, i.e., *R_L_ = σ*/Δ*p_L_* and *R_R_ = σ*/Δ*p_R_*. Note that the pressure difference across the left interface is larger than that across the right interface, i.e., Δ*p_L_* > Δ*p_R_*. This indicates a larger curvature radius of the right interface than that of the left interface, i.e., *R_R_* > *R_L_*, which is consistent with the observation in Figure 4c. At T3, the pressure difference across the droplet interface rises significantly, i.e., Δ*p_L_ =* 4.86 and Δ*p_R_* = 4.75, suggesting a decreased curvature radius of the droplet interface. This may result from the squeezing effect on the water droplet by the medium oil fluid. At T4, the pressure difference across the right interface reduces significantly to Δ*p_R_* = 4.27, which is smaller than that across the left interface Δ*p_L_ =* 4.65. The pressure of the medium oil fluid on the right of the droplet is larger than that on the left of the droplet, i.e., *p_oR_* > *p_oL_*. At T5, the plateau of the high pressure moves towards the negative *x*-axis. This suggests that the droplet travels into the left channel.

**Figure 6:**
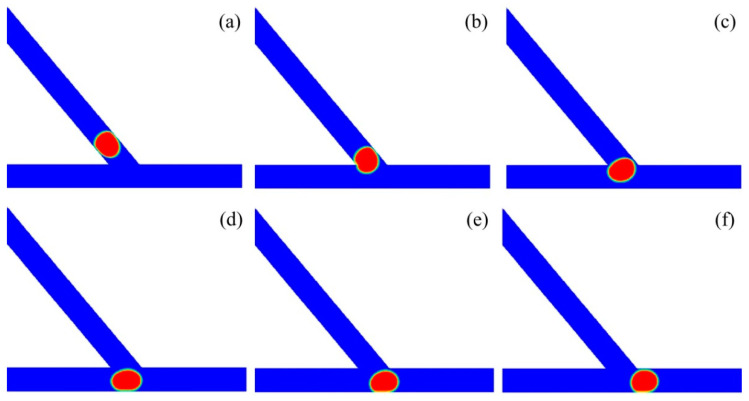
Passive sorting of small droplet into the right channel (*θ* = 50°, *U* = 20 mm/s, *V*_d_*/w*^3^ = 0.6). (**a**) T0, (**b**) T1 = T0 + 0.6, (**c**) T2 = T0 + 1, (**d**) T3 = T0 + 1.6, (**e**) T4 = T0 + 2.8, (**f**) T5 = T0 + 3.6.

Figure 6 presents the process of small droplet sorting into the right channel of the asymmetric junction. The asymmetric junction had an angle of *θ* = 50° and the inlet velocity was set to *U* = 20 mm/s, with the corresponding capillary number of *Ca* = 0.032. The droplet had a non-dimensional volume of *V*_d_*/w*^3^ = 0.6. The time propagates from T0 to T5. The droplet starts to enter into the branching junction at the time of T0. At T1, the head of the droplet enters into the junction, with the left side of the droplet attached the left corner of the acute junction. At T2, the majority of the droplet moves into the branching channel. Additionally, further at T3, the droplet completely enters into the acute junction. At T4, the droplet moves rightwards, producing a tunnel between the left part of the droplet and the left corner of the acute junction. At T5, the droplet completely moves into the right channel.

**Figure 7:**
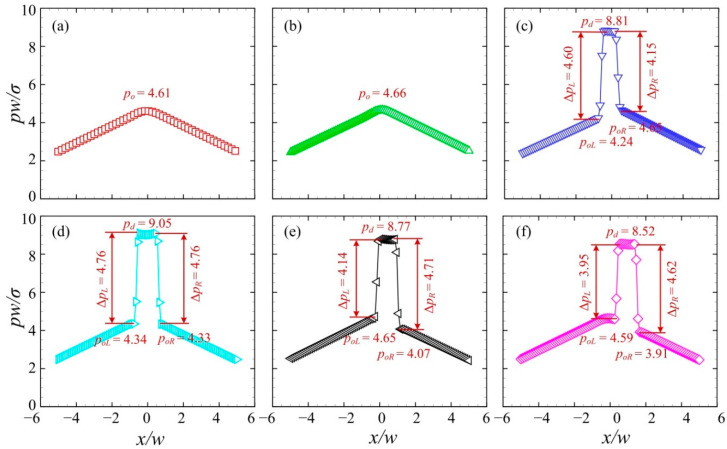
Pressure distributions along the centreline of the branching channels when the droplet is sorted into the right channel at *V*_d_*/w*^3^ = 0.6 (*θ* = 50°, *U* = 20 mm/s). (**a**) T0, (**b**) T1, (**c**) T2, (**d**) T3, (**e**) T4, (**f**) T5.

Figure 7 shows the pressure distributions along the centreline of the branching channels when the droplet moves towards the right channel. The sequential times correspond to those in Figure 6. At T0 and T1, the pressure exhibits a hill-like distribution with the largest value at the center before the droplet arrives at the centreline of the branching chancel. At T2, the pressure of the droplet increases substantially to *p_d_* = 8.81, forming a plateau of high pressure at the center. Note that the Laplace pressure across the left interface Δ*p_L_* is larger than that across the right interface Δ*p_R_*. This suggests a larger curvature radius of the right interface than that of the left interface. At T3, the pressure difference across the left and right interfaces rises to Δ*p_L_ =* Δ*p_R_* = 4.76, indicating an equivalent curvature radius of both interfaces. At T4, the pressure difference across the left interface decreases substantially to Δ*p_R_* = 4.14, which is smaller than that at across the left interface Δ*p_L_ =* 4.71. The pressure of the medium oil fluid on the left of the droplet is higher than that on the right of the droplet, i.e., *p_oL_* > *p_oR_*. At T5, the plateau of the high pressure moves towards the positive *x*-axis. This suggests that the droplet travels into the right channel.

### 3.2. Velocity-Based Droplet Sorting

The velocity-based droplet sorting can be achieved within the asymmetric acute junction. The droplet with large velocity enters into the left channel, while the droplet with small velocity moves into the right channel. Both the subsequent stages of the droplet sorting and corresponding pressure distribution are examined in this section.

**Figure 8:**
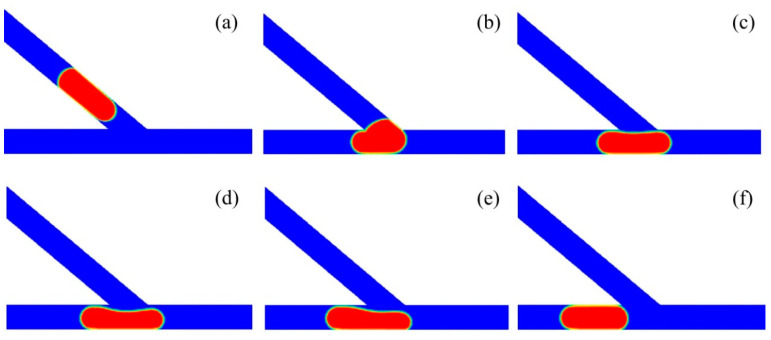
Passive sorting of droplet into the left channel at a relatively large velocity of *U* = 10 mm/s (*θ* = 40°, *V*_d_/*w*^3^ = 2). (**a**) T0, (**b**) T1 = T0 + 2.55, (**c**) T2 = T0 + 3.25, (**d**) T3 = T0 + 3.75, (**e**) T4 = T0 + 4.65, (**f**) T5 = T0 + 6.05.

Figure 8 shows the subsequent stages of a droplet sorting into the left channel at a relatively large velocity. The time propagates from T0 to T5. The acute junction had an angle of *θ* = 40° and the droplet had a volume of *V*_d_*/w*^3^ = 2. The inlet velocity was set to *U* = 10 mm/s and the corresponding capillary number was *Ca* = 0.016. Initially, the droplet moves in the main channel at T0. Then, the droplet starts to enter into the acute junction. At T1, the majority of the droplet enters into the junction, and a left “finger” occurs in the left channel. Further at T3, a right “finger” is evident in the right channel, and the droplet completely enters into the acute junction. At T3, the rear of the droplet becomes concave at the central region of the acute junction. At T4, the right part of the droplet is squeezed by the medium fluid, resulting in a small curvature radius of the right interface of the droplet and a tunnel between the right part of the droplet and the right corner of the junction. The medium fluid moves to the right through the tunnel and the droplet starts to move leftwards. Finally, at T5, the droplet completely travels into the left channel.

**Figure 9:**
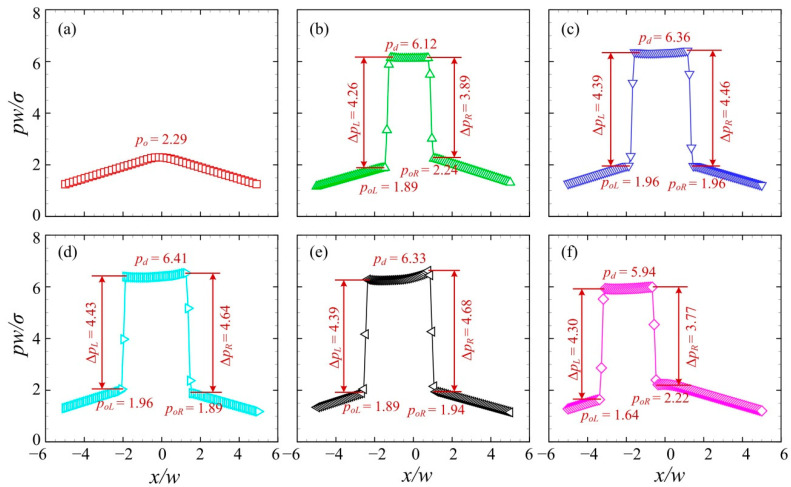
Pressure distributions along the centreline of the branching channels when the droplet is sorted into the left channel at *U* = 10 mm/s (*θ* = 40°, *V*_d_/*w*^3^ = 2). (**a**) T0, (**b**) T1, (**c**) T2, (**d**) T3, (**e**) T4, (**f**) T5.

**Figure 10:**
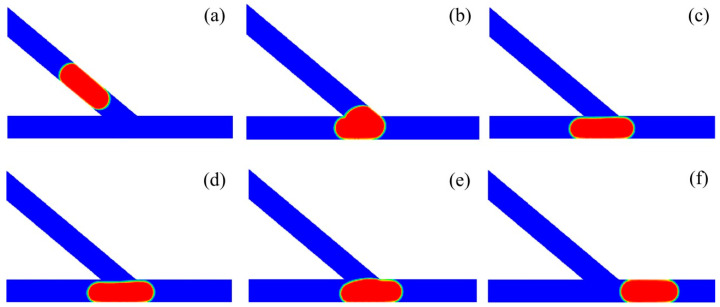
Passive sorting of droplet into the right channel at the relatively small velocity of *U* = 4 mm/s (*θ* = 40°, *V*_d_/*w*^3^ = 2). (**a**) T0, (**b**) T1 = T0 + 2.56, (**c**) T2 = T0 + 3.52, (**d**) T3 = T0 + 4, (**e**) T4 = T0 + 4.48, (**f**) T5 = T0 + 8.

Figure 9 presents the pressure distributions along the centreline of the branching channels when the droplet with a relatively large velocity moves to the left channel. The junction angle was *θ* = 40° and the initial volume of the droplet was *V*_d_/*w*^3^ = 2. The inlet velocity was set to *U* = 10 mm/s. The time series from T0 to T5 correspond to those in Figure 8. The distribution of the dimensionless pressure at T0 was used for the reference. At T1, the pressure at the central region of the junction rises substantially, forming a plateau with a value of *p_d_* = 6.12. The pressure difference across the left interface is larger than that across the right, i.e., Δ*p_L_* > Δ*p_R_*. Note that the pressure of the medium oil fluid at the left side of the droplet *p_oL_* is smaller than that at the right side *p_oR_*. This can be ascribed to the left “finger” formed at the left side (see Figure 10b). At T2, the droplet pressure increases to *p_d_* = 6.36 and pressure difference across the droplet interface rises to ~4.40. At T3, the pressure difference across the right interface goes up to Δ*p_R_* = 4.64, indicating a reduced curvature radius of the right interface of the droplet. At T4, the pressure difference across the right interface increases slightly, while that across the left interface decreases. Additionally, both the left and right interfaces of the droplet move towards the negative *x*-axis. Finally, at T5, a more evidently leftward movement of the droplet interfaces can be observed.

Figure 10 presents the subsequent stages of the droplet sorting into the right channel at a relatively small velocity. The acute junction had an angle of *θ* = 40° and an inlet velocity of *U* = 4 mm/s, with the corresponding capillary number of *Ca* = 0.0064. The non-dimensional volume of the droplet was set to *V*_d_/*w*^3^ = 2. The time T0 was used for the reference, before moving into the acute junction. At T1, the droplet enters into the junction, forming a left “finger” in the left channel. At T2, the droplet completely travels into the junction. At T3, a small tunnel occurs between the left side of the droplet and the left corner of the acute junction. Then, the droplet travels rightwards at T4 and finally enters into the right channel at T5.

**Figure 11:**
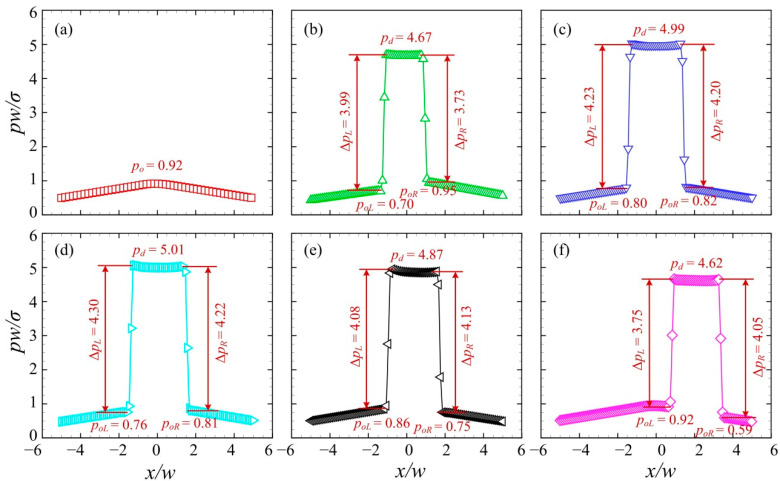
Pressure distributions along the centreline of the branching channels when the droplet is sorted into the right channel at *U* = 4 mm/s (*θ* = 40°, *V*_d_/*w*^3^ = 2). (**a**) T0, (**b**) T1, (**c**) T2, (**d**) T3, (**e**) T4, (**f**) T5.

**Figure 12:**
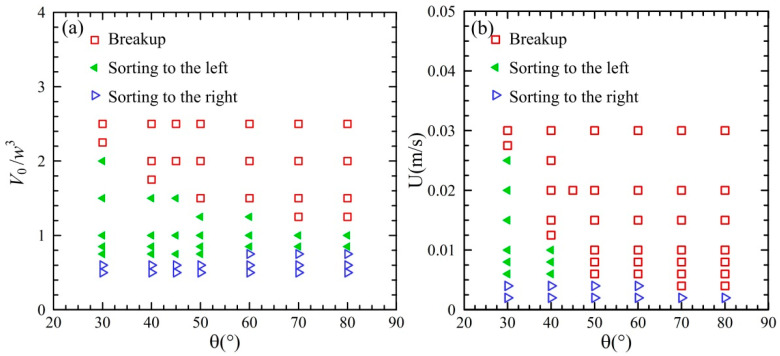
Phase regime of droplet sorting in the asymmetric acute junctions: (**a**) *U* = 20 mm/s and (**b**) *V*_d_/*w*^3^ = 2.

Figure 11 presents the pressure distributions along the centreline of the branching channels when the droplet moves to the left channel at a relatively small velocity. The time series from T0 to T5 correspond to those in Figure 10. The pressure distribution at T0 was used for the reference, with a maximum oil pressure of *p_o_* = 0.92. At T1, the pressure at the central region of the junction rises sharply to *p_d_* = 4.67, due to the entry of the droplet into the junction. The pressure of the medium oil fluid at the left side of the droplet *p_oL_* is smaller than that at the right side *p_oR_*. This is due to the left “finger” formed in the left channel (see Figure 12b). At T2, the pressure at the central region increases to *p_d_* = 4.99 and pressure difference across the droplet interface rises to ~ 4.20, suggesting a reduced curvature radius of the droplet in the branching channels. At T3, the pressure difference across the left interface rises to Δ*p_L_* = 4.30, while that across the right interface remains roughly unchanged. This indicates that the left interface of the droplet has a reduced curvature radius. At T4, both the left and right interfaces of the droplet travel towards the positive *x*-axis. Finally, at T5, the rightward movement of the droplet interfaces is more visible.

### 3.3. Phase Regime of Droplet Behavior in the Acute Junction

The phase regime of the droplet sorting in the acute junction was examined, as shown in Figure 12. The angle *θ* of the acute junction varied from 30° to 80°. The non-dimensional volume of the droplet was within in the range of *V*_d_/*w*^3^ = 0.5 ~ 2.5 and the velocity was from 4 mm/s to 20 mm/s. The dependence of the phase regime on the droplet volume and the junction angle is shown in Figure 12a. The velocity was fixed at *U* = 20 mm/s. For the droplet volume over *V*_d_/*w*^3^ = 1.0 ~ 2.5, the droplet undergoes a transition from breakup to sorting left, with the decrease in droplet volume. For the droplet volume over *V*_d_/*w*^3^ = 0.5 ~ 1.0, a transition from sorting left to sorting right can be observed with the reduction in droplet volume. The junction angle has a significant effect on the transition between breakup and sorting left, but not between sorting left and sorting right. The dependence of the droplet behavior on the velocity and the junction angle is presented in Figure 12b. The non-dimensional volume of the droplet is of *V*_d_/*w*^3^ = 2. For the junction angle *θ* = 30° and 40°, the droplet exhibits a transition from breakup to sorting left and then to sorting right, with the reduction in the velocity. For the junction angle *θ* = 50° ~ 80°, the transition from breakup to sorting right can be observed with the decrease in the velocity. Therefore, droplet breakup occurs at large volume or high velocity, while droplet sorting is present at relatively small volume and low velocity. A careful consideration should be made to fulfil the droplet sorting with asymmetric acute junctions.

## 4. Discussion

**Figure 13:**
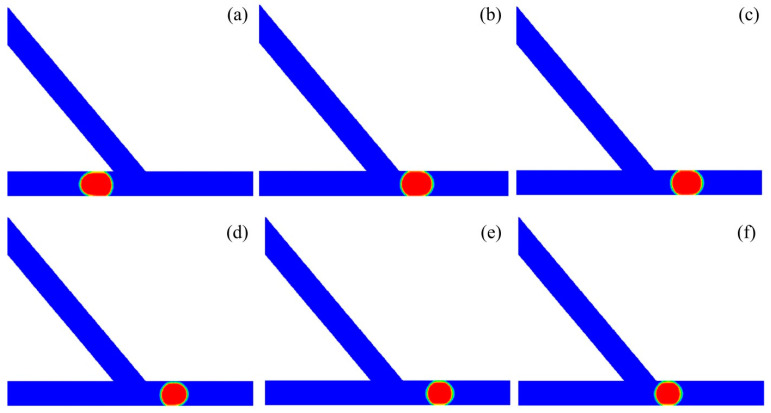
Droplet sorting in the asymmetric acute junction with the angle of *θ* = 50° at various interfacial tensions. (**a**) *V*_d_/*w*^3^ = 0.85, σ = 3 mN/m, (**b**) *V*_d_/*w*^3^ = 0.85, σ = 7 mN/m, (**c**) *V*_d_/*w*^3^ = 0.85, σ = 12 mN/m, (**d**) *V*_d_/*w*^3^ = 0.6, σ = 3 mN/m, (**e**) *V*_d_/*w*^3^ = 0.6, σ = 7 mN/m 4, (**f**) *V*_d_/*w*^3^ = 0.6, σ = 12 mN/m.

In most real droplet-based microfluidics, the fluorinated oil FC40 is one of commonly used medium fluid and 1–5% PEG-PFPE is used as surfactant. To examine whether the proposed method can be used in real experiments, we further investigated the volume-based droplet sorting in the acute junction with an angle of *θ* = 50°. In these simulations, FC40 was used as the medium fluid, with the density and viscosity of *ρ*_m_ = 1850 kg/m^3^ and μ_m_ = 4.1×10^−3^ Pa·s, respectively. Following the experimental studies of droplet flow in Refs. [74,75], in which 1–5% PEG-PFPF was used for the surfactant, three values of interfacial tensions were chosen, i.e., σ = 3, 7, and 12 mN/m. The channel walls were set to be hydrophobic with a contact angle of 150°. Figure 13 presents the droplet sorting behavior at different interfacial tensions. It is observed that the droplet exhibits different sorting behavior at different interfacial tensions. The droplet with the volume of *V*_d_/*w*^3^ = 0.85 enters into the left channel at the interfacial tension of σ = 3 mN/m while moving into the right channel at σ = 7 and 12 mN/m. Moreover, the droplet undergoes a transition of sorting left to sorting right with the reduction in *V*_d_/*w*^3^ from 0.85 to 0.6 at σ = 3 mN/m. In contrast, the droplets with the volume of *V*_d_/*w*^3^ = 0.85 and 0.6 are all sorted to the right channel at σ = 7 and 12 mN/m. These facts indicate that the interfacial tension has a great impact on the sorting behavior. It can be inferred that the boundary between sorting left and sorting right in the phase regime is different at various interfacial tension. In spite of this, these results show that the proposed asymmetric acute junction could be used for droplet sorting in real applications of droplet-based microfluidics.

Many methods have been developed for droplet sorting in recent years. For example, acoustic methods can achieve the on-demand sorting of the droplets [31,32]. However, one disadvantage of acoustic methods is that their operation requires expensive and complex equipment, such as function generators, voltage amplifiers, signal processors, and acoustic transducers [36]. In contrast, the proposed passive sorting method only relies on the geometry of asymmetric acute junctions. It has the advantages of simple-fabrication and cost-effective. Additionally, a high-throughput sorting could be achieved using a combination of a massive number of acute junctions in one microfluidic chip.

## 5. Conclusions

This work presented numerical investigations on the droplet sorting in the asymmetric junctions. We found that the asymmetric acute junction could be used for passive droplet sorting. Both volume-based and velocity-based droplet sortings can be achieved in the asymmetric acute junctions. For volume-based droplet sorting, large droplets are sorted to the left channel, while small droplets are sorted to the right channel. For velocity-based droplet sorting, fast droplets are sorted to the left channel, while slow droplets are sorted to the right channel. The physical mechanism behind the droplet sorting is associated with the pressure distribution surrounding the droplet when the droplet enters into the junction. The droplet can be sorted to the left if the accumulated pressure at the rear of the droplet could open a tunnel between the droplet and the obtuse corner of the asymmetric junction. A phase diagram of the droplet sorting is presented to unveil the dependence of the droplet sorting on the droplet volume and the velocity at various junction angles. Further work is required to experimentally investigate passive droplet sorting using asymmetric acute junction and systematic studies on the effect of the junction geometry on the sorting accuracy of the droplets with different sizes and deformability. Moreover, this sorting method based on the acute junction could be integrated with other sorting methods to further improve the sorting performance.
